# The provision of generalist and specialist palliative care for patients with non-malignant respiratory disease in the North and Republic of Ireland: a qualitative study

**DOI:** 10.1186/s12904-017-0220-1

**Published:** 2017-07-11

**Authors:** Clare Mc Veigh, Joanne Reid, Philip Larkin, Sam Porter, Peter Hudson

**Affiliations:** 1 School of Nursing and Midwifery, Queen’s University Belfast, Belfast, Northern Ireland; 2 School of Nursing, Midwifery and Health Systems, University College Dublin, Dublin, Ireland; 3 Department of Social Sciences and Social Work, Bournemouth University, Dorset, UK; 4 Centre for Palliative Care, c/o St Vincent’s University Hospital and University of Melbourne, Melbourne, Australia

**Keywords:** Palliative care, Non-malignant respiratory disease, COPD, Interstitial lung disease, Bronchiectasis, Healthcare Professionals, Qualitative research and bereaved carers

## Abstract

**Background:**

Previous research and key guidelines have suggested potential models of palliative care for patients with COPD and interstitial lung disease. However, these recommendations are often not effectively implemented in clinical practice and are void of guidance regarding palliative care for patients with bronchiectasis, another form of non-malignant respiratory disease. The aim of this research was to explore generalist and specialist palliative care service provision for people with non-malignant respiratory disease in the North and Republic of Ireland.

**Methods:**

Qualitative study involving a convenience sample of 17 bereaved carers and 18 healthcare professionals recruited from 2 rural and 2 urban sites on the Island of Ireland. Data collection consisted of semi-structured interviews with carers of patients with COPD, interstitial lung disease or bronchiectasis who had died 3–18 months previously; and 4 focus groups with healthcare professionals. Data analysed using thematic analysis.

**Results:**

Findings highlighted the lack of a clear model of holistic care delivery for patients with non-malignant respiratory disease and illuminated the varying levels of palliative care provision this client group experienced. Additionally, ambiguity amongst healthcare professionals regarding prognostication illuminated the importance of the provision of palliative care being based on patient need, not prognosis. This research developed a potential model of palliative care which may help healthcare professionals introduce palliative care, and specialist respiratory care, early in the disease trajectory of non-malignant respiratory disease, whilst also encouraging the involvement of specialist palliative care for complex symptom management.

**Conclusion:**

This research provides an important insight into a potential model of palliative care for people with non-malignant respiratory disease, inclusive of bronchiectasis. However, the feasibility of integrating this model into clinical practice requires further exploration.

**Electronic supplementary material:**

The online version of this article (doi:10.1186/s12904-017-0220-1) contains supplementary material, which is available to authorized users.

## Background

The World Health Organisation [1] highlighted that in 2012 more than 3 million people died of COPD, which globally represented 6% of all deaths that year. Although confirmability data are not available it is estimated that the extent of other non-malignant respiratory diseases (NMRDs), such as interstitial lung disease (ILD) and bronchiectasis, are a growing global health problem [2]. Within Europe, it is estimated that mortality rates of ILD are approximately 2.5 per 100,000 people per year [3]. In the United Kingdom (UK) it was highlighted that there were 1.68 deaths from bronchiectasis per 100,000 population in 2007, with the mortality rate increasing by approximately 3% per year [4]. Patients with NMRD should receive a palliative approach to their care, even if they are still actively receiving treatment to slow or stabilise their illness [5]. Although NMRD is on the increase internationally and nationally evidence suggested that patients with NMRD often do not receive optimum holistic care [6], or the same standards of palliative care as patients with malignant respiratory disease [7, 8]. However this research [7, 8] focused on patients with a diagnosis of COPD and their carers, and not those with ILD or Bronchiectasis.

The palliative care provided by healthcare professionals (HCPs) that specialise in this area is known as specialist palliative care, and these professionals provide palliative expertise, services and resources [9]. Palliative care delivered by HCPs who are not classified as specialist palliative care providers is known as generalist palliative care [10]. There are several key strategic drivers within Northern Ireland (NI) [11, 12] and the Republic of Ireland (ROI) [13–15] that have highlighted the need for generalist and specialist palliative care provision for patients with NMRD. However, there is a lack of consensus amongst national [16] and international [17–22] guidelines regarding palliative care for patients with NMRD and their carers. These strategic documents are also void of guidance regarding palliative care for patients with bronchiectasis. Specialist respiratory HCPs also have a key role in the provision of generalist palliative care to patients with NMRD [16]. Specialist respiratory services for patients with a malignant or non-malignant respiratory condition consist of HCPs specialised in respiratory care that include respiratory nurse specialists, respiratory consultants and allied health professionals. The provision of specialist respiratory services for patients with NMRD varies throughout the North and Republic of Ireland, with these services being more readily available in the primary care setting in NI [11] in comparison to the ROI [23]. The majority of specialist respiratory services in the ROI are based in the secondary care setting. The coordination and availability of specialist respiratory, specialist palliative care and generalist palliative care services differ across the North and Republic of Ireland, especially within the primary care setting.

### Aim

The aim was to explore generalist and specialist palliative care service provision for people with NMRD in the North and Republic of Ireland.

## Methods

### Design

The design of this study involved two methods of data collection: semi- structured interviews with bereaved carers of patients with NMRD, and focus groups with HCPs involved in their care. Due to the exploratory nature of this research a broad interpretivist approach was utilised by the researcher. Interpretivist researchers believe that by interacting with the world around them people attach their own meanings and values to their experiences, therefore broad interpretivism attempts to explore this to provide a deeper understanding of the topic being researched [24]. Something that must be considered in qualitative research when discussing the research process is reflexivity. The main researcher (C.V) was a registered nurse with 10 years experience caring for patients with palliative care needs.

### Settings and participants

**Fig. 1:**
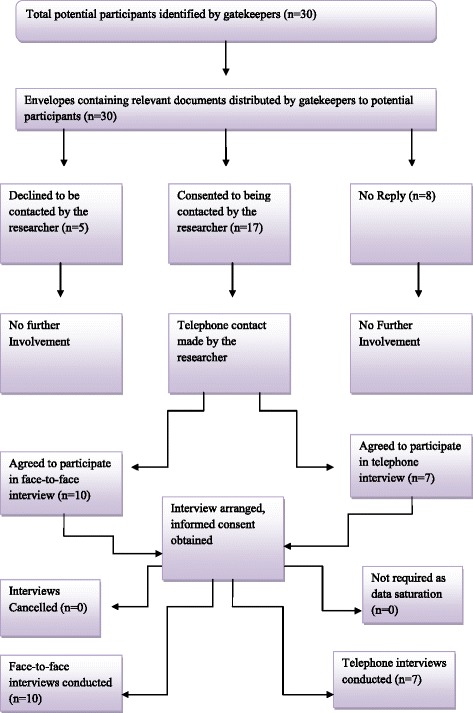
Flowchart of recruitment and retention for semi-structured interviews with bereaved carers

**Fig. 2:**
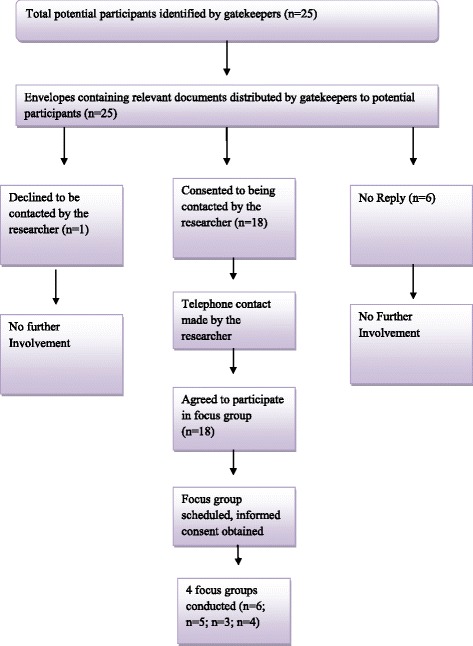
Flowchart of recruitment and retention for focus groups with healthcare professionals

A total of 17 bereaved carers and 18 HCPs were recruited from 1 rural and 1 urban site in NI, and 1 rural and 1 urban site in the ROI using convenience sampling. In NI, the research involved bereaved carers and HCPs in the Northern Health and Social Care Trust (NHSCT) and the Belfast Health and Social Care Trust (BHSCT). In the ROI, bereaved carers and HCPs were recruited from Letterkenny, Co. Donegal and Dublin. Belfast is acknowledged as an urban area in NI [25] and Dublin is recognised as an urban location in the ROI [26]. The NHSCT covers a large rural area in NI [27] and Letterkenny is part of a rural county in the ROI [28]. Figures 1 and 2 illustrate the recruitment process. Tables 1 and 2 provide the sociodemographic details of interview and focus group participants. As noted within Table 1, the majority of carers were female (*n* = 14) and all carers were family members. Eligibility criteria for participation in the semi-structured interviews identified bereaved carers, ascertained by a respiratory nurse specialist (RNS) as the main carer, of people with NMRD who had died 3 to 18 months previously. Bereaved carers were identified from the patients’ documentation, and recruited, by the RNS in both the secondary and primary care setting. This group of participants were chosen as the researcher felt they would not only give an insight into the patient’s palliative care throughout their illness, but also their own experiences as a carer. Bereaved carers were also used as proxies for the patient as they were able to provide a post-death account that also took into consideration the palliative care that was provided to the patient at the end of life [29]. The period of time was chosen as although it was not straight after the death of the participant’s relative, it was within a time frame that allowed them to remember their experience and also reduced trauma to the participant. Carers under 18 years of age, who did not speak English or were too distressed to take part in a research study, were excluded. Eligibility criteria for the focus groups included HCPs involved in the generalist and specialist palliative care of patients with NMRD, in the primary and secondary care setting. Permission to access staff was sought from the various relevant heads of each department e.g. director of nursing, respiratory consultant etc., in the areas involved in the study and they also aided recruitment.

**Table 1:** Bereaved carer participant profiles

Participants of the semi-structured interviews	
Gender	Women: 14 bereaved carers
	Men: 3 bereaved carers
Age	Mean: 58.2 years
	Median: 55 years
	Range: 41–81 years
Relation to patient	Daughter (*n* = 5)
	Son (*n* = 2)
	Wife (*n* = 7)
	Partner (*n* = 1)
	Daughter in-law (*n* = 1)
	Nephew (*n* = 1)
Patient and carer living together	Yes (*n* = 10)
	No (*n* = 7)
Occupation	Residential home manager (*n* = 1)
	Unemployed (*n* = 3)
	Retired (*n* = 6)
	Classroom Assistant (*n* = 1)
	Housewife (*n* = 3)
	Probation Officer (*n* = 1)
	Porter (*n* = 1)
	Health and Safety Officer (*n* = 1)
Time post bereavement	Mean: 7 months
	Median: 6 months
	Range: 4–17 months
Area	Northern Ireland (*n* = 10)
	Republic of Ireland (*n* = 7)
	Rural (*n* = 8)
	Urban (*n* = 9)
Diagnosis	COPD (*n* = 12)
	Interstitial Lung Disease (*n* = 4)
	Bronchiectasis (*n* = 1)
Interview Type	Face to Face (*n* = 10)
	Telephone (*n* = 7)

**Table 2:** Healthcare professional participant profiles from primary and secondary care setting

Participant	Occupation	Years of Experience in Current Role
Focus Group 1 (Urban, NI)		
OT1	Specialist palliative care Occupational Therapist	20 years
RN1	Respiratory Nurse Specialist	5 years
PCN1	Palliative Care Nurse Specialist	7 years
PCC1	Specialist palliative care Consultant	13 years
PCN2	Specialist palliative care Nurse Consultant	23 years
RC1	Respiratory Consultant	15 years
Focus Group 2 (Rural, NI)		
RN2	Respiratory Nurse Specialist	13 years
RN3	Respiratory Ward Manager	10 years
RC2	Respiratory Consultant	5 years
RN4	Respiratory Nurse Specialist	5 years
PCN3	Palliative Care Inpatient unit Deputy Ward Manager	32 years
Focus Group 3 (Rural, ROI)		
RN5	Respiratory Clinical Nurse Specialist	6 years
PCN4	Palliative Care Clinical Nurse Specialist	11 years
PH	Physiotherapist	11 years
Focus Group 4 (Urban, ROI)		
RN6	Respiratory Clinical Nurse Specialist	20 years
RN7	Respiratory Clinical Nurse Specialist	12 years
RN8	Respiratory Deputy Ward Manager	6 years
PCN5	Palliative Care Clinical Nurse Specialist	12 years

### Data collection

Semi-structured interviews (Additional file 1: Box 1) were conducted in bereaved carers’ homes (*n* = 10), or over the telephone (*n* = 7). The choice of telephone or face-to-face was offered to participants to allow them to choose an interview method that was most convenient for them. Research has previously highlighted that bereaved family members interviewed over the telephone did not report any concerns or limitations associated with this data collection method [30, 31]. Interviews ranged from 22 to 80 min in length, with an average of 50 min. Healthcare professional focus groups (*n* = 4) (Additional file 2: Box 2) were conducted with participants from the primary and secondary care setting at each area involved in the study. Focus groups ranged from 68 to 81 min in length, with an average of 74 min. All interviews and focus groups were digitally recorded and transcribed verbatim. Interviews and focus groups were conducted by one author (C.V) who had appropriate training on conducting qualitative research. In order to aid the accurate recording of the focus groups, an additional member of the research team fulfilled the role of note taker (J.R). Data collection was completed when it was noted by the researcher that no new themes or information were emerging from the data.

### Analysis

Data were analysed by adopting King and Horrock’s [32] approach to thematic analysis. The qualitative research software package NVivo was used to allow for the management of the dataset, whilst also facilitating an increased focus on effectively analysing the data [33]. Initial data analysis consisted of the exploration of each interview and focus group to develop descriptive themes. Descriptive themes generate a list of emerging categories, or topics, from the data but do not offer an interpretation of their meanings [34]. Analysis then progressed to focusing on the interpretation of the meaning of the data [32] through grouping together the descriptive themes to highlight emerging interpretative themes. Finally, overarching themes were developed by linking all the interpretative themes that had been established. Each transcription was initially analysed by C.V and then reviewed separately by the remaining authors (P.H, J.R, P.L and S.P). All themes and transcripts were then discussed collaboratively by the research team for verification of the themes generated, and agreement was reached regarding the final themes. Preliminary findings were also presented at various academic conferences and clinical meetings which provided further opportunities for feedback and critical discussions.

### Ethics

All participation was voluntary with full written, and verbal, informed consent. Ethical approval was received from the Office of Research and Ethics Committee Northern Ireland (ORECNI, Reference: 11/NI/0166) and governance permission from the Northern Health and Social Care Trust (Reference: NRP11–0264/09), Belfast Health and Social Care Trust (Reference:11080JR-AS), Letterkenny and Dublin.

#### Semi-structured interviews findings

Analysis of the carers’ interviews identified the key overarching theme: *lack of consistency in palliative care delivery,* developed from the interpretative themes outlined below and illustrated through participants’ quotes.

#### Lack of consistency in palliative care delivery

The overarching theme of lack of consistency in palliative care delivery identified interpretative themes of ‘*variations in the delivery of specialist palliative care’* and *‘variations in the delivery of palliative care from specialist respiratory services.’*

### Variations in the delivery of specialist palliative care

Amongst the 17 carers that were interviewed, 6 stated that the patient they cared for had received specialist palliative care input. Specialist palliative care was viewed by participants as care being delivered by HCPs who identified themselves as being specifically trained in delivering palliative care. The remaining 11 carers stated that, to the best of their knowledge, the patient had not received input from a specialist palliative care provider. Out of the 6 who perceived that the patient had received specialist palliative care, 2 had a diagnosis of ILD and 4 had a diagnosis of COPD. Carers who reported the patient as having received specialist palliative care expressed that these services were only introduced when the patient was nearing the end of their life:

*“[Specialist] Palliative care was only introduced the week he died so that didn’t even really get off the ground.”* (BC17, p4).

The absence of specialist palliative care providers was also recognised and discussed by some carers who highlighted that they would have preferred these services to be involved in patients’ care:

*“We would have liked palliative care were you would have a qualified nurse coming in who knew exactly what was happening here. I feel we would have benefited greatly from that. But we didn’t have that.”* (BC1, p13).

Carers’ experiences highlighted their perceptions that the early introduction of specialist palliative care may have been of benefit to the patient with NMRD.

### Variations in the delivery of palliative care from specialist respiratory services

The provision of palliative care to patients with NMRD by specialist respiratory services was also illuminated by carers. In the ROI services from a RNS were available in the secondary care setting and not the primary care setting. Carers in the ROI perceived that the receipt of services from a RNS would have been of benefit to the patient in the community:

*“I think maybe if he had someone coming to him once a month or every three weeks like a respiratory nurse, I would have liked them to be more involved but they don't have services like that here.”* (BC3, p2).

Carers within the ROI perceived that patients with NMRD received adequate specialist respiratory care for their palliative needs when in hospital, but this care was not continued when the patient was discharged back into the community:

*“Well she didn’t really have any care at home apart from when she took sick and went into hospital. Then she was seen by doctors and nurses but nothing at home. She would have seen the respiratory nurse but that was only up at the hospital.”* (BC10, p1).

In contrast to the views expressed by carers in the ROI, in NI perceptions were that patients with NMRD had sufficient input from the RNS in the primary care setting:

*“Like if it was anytime during the day I would have just rang up the respiratory nurse and there was always someone there to help me.”* (BC14, p4).

Variances in the provision of specialist respiratory care services for patients with NMRD were therefore indicated by the accounts provided by carers.

#### Focus group findings

Two overarching themes emerged from analysis of the HCPs’ interviews*: barriers to providing appropriate palliative care* and *future direction of palliative care for patients with NMRD.* Overarching themes originated from the interpretative themes outlined below and illustrated through participants’ quotes.

#### Barriers to providing appropriate palliative care

The overarching theme of barriers to providing appropriate palliative care identified interpretative themes of *‘lack of prognostic certainty’* and *‘Lack of understanding of the role of palliative care in relation to patients with NMRD.’*

### Lack of prognostic certainty

Across the North and ROI, varying HCPs perceived that uncertainty regarding the trajectory of NMRD inhibited the timely provision of palliative care to this client group:

“*I think that the disease [NMRD] can be palliative from a much earlier stage and I am not sure how good we are at that, I think someone could be palliative from their first referral to the clinic and they might take five years to die. Prediction is often difficult; it is a little bit easier with pulmonary fibrosis as the line of trajectory tends to be more acute. And very difficult in Bronchiectasis, it’s only after several runs of treatment that you realise that this isn’t working. I think we often kind of look back and think could I have spotted something six months ago? I always ask myself are we late into the game of palliation?”* (RC1, Respiratory Consultant, p4).

*“With lung cancer patients you can give a rough prognosis and know when to introduce palliative care. With patients with COPD there is no definitive timeline and they can be up and down so there is no obvious point where you know to definitely involve palliative care so that is the big issue really.”* (RN8, Respiratory Deputy Ward Manager, p8).

Difficulties regarding prognostication due to the clinical manifestations of NMRD, particularly COPD and bronchiectasis, were highlighted as resulting in the delayed introduction of palliative care in the disease trajectory.

### Lack of understanding of the role of palliative care in relation to patients with NMRD

Healthcare professionals’ perceived that specialist palliative care was often still only associated with malignant disease, and therefore HCPs did not recognise the important role it had in NMRD:

*“You know awareness is improving but there still are professionals who are surprised and will say, “That patient is respiratory they haven’t got cancer so why are you seeing them?” So there is still a gap in the awareness even among professionals.”* (PCN4, Palliative Care Nurse Specialist, p5).

Participants in NI and the ROI additionally suggested that specialist palliative care providers viewed patients with a malignant diagnosis as having priority over those with NMRD:

*“You know I have had fights and struggles with the specialist palliative care team because they see cancer as a priority and not respiratory and they don’t understand that these people have palliative needs too and therefore they wouldn’t prioritise them as much for the Marie Curie sit in service.”* (RN2, Respiratory Nurse Specialist, p4).

The views expressed by HCPs in the ROI additionally indicated an inequality of generalist and specialist palliative care service provision for patients with ILD or bronchiectasis, in comparison to COPD:

*“Bronchiectasis patients are left to self -manage at home and I would say that the COPD patients are getting a better [specialist and generalist] palliative care service than the other cohorts [ILD and bronchiectasis].”* (RN7, Respiratory Nurse Specialist, p9).

*“I suppose from what I have seen it is mostly NMRD patients with COPD that get seen by specialist palliative care and I haven’t had any bronchiectasis patients that have been seen or referred to specialist palliative care that I am aware of.”* (PH, Physiotherapist, p8).

Healthcare professionals’ accounts illuminated how a perceived lack of understanding amongst HCPs in relation to the role of palliative care for patients with NMRD, created a barrier to optimal service provision.

#### Future direction of palliative care for patients with NMRD

The overarching theme of future direction of palliative care for patients with NMRD identified interpretative themes of *‘who will provide palliative care?’* and *‘the presence of a model for palliative care delivery in NMRD.’*

### Who will provide palliative care?

When discussing the future provision of palliative care to patients with NMRD in the North and ROI, HCPs presented varied views on who should be involved in the delivery of palliative care to this client group. Some medical and nursing HCPs expressed that the involvement of specialist palliative care providers was a necessity for patients with NMRD:

*“I would rather if I had a condition like NMRD I would probably prefer to have a specialist palliative care consultant than a generalist respiratory physician that has had some experience in palliative care.”* (RC2, Respiratory Consultant, p12).

Allied and nursing HCPs perceived that increased involvement of specialist palliative care services equated in reduced numbers of emergency admissions for patients with NMRD:

*“And I know it is far and few between but the [NMRD] patients who have seen specialist palliative care maybe not coming in as much to accident and emergency as they are not panicking and so therefore specialist palliative care can make a big difference.”* (PH, Physiotherapist, p6).

*“I think nursing homes sometimes send (NMRD) patients in (to hospital) without really getting someone to see why they need to be sent into hospital because they do tend to sit in accident and emergency for hours unnecessarily. You know I think if the hospice nurse did go in to have a look then a lot of the admissions could be avoided.”* (PCN3, Palliative Care Inpatient Unit Deputy Ward Manager, p10).

Participants from the varying healthcare professions additionally conveyed that specialist palliative care involvement was necessary when a patient with NMRD developed complex symptom needs:

*“I know my limitations in terms of symptomatically what can the girls [specialist palliative care nurses] bring in that we [RNS] perhaps wouldn’t even consider and therefore I think that specialist palliative care is required on that account.”* (RN7, Respiratory Nurse Specialist, p8).

However HCPs additionally illuminated that the delivery of holistic care to patients with NMRD was part of the role of all generalist palliative care providers:

*“I think that in some of these NMRD patients the fact that they have a chronic disease may not mean that they need specialist palliative care but certainly a palliative approach is needed and is all our responsibilities regardless of our qualifications.”* (PCN4, Palliative Care Nurse Specialist, p5).

Healthcare professionals’ accounts highlighted mixed perspectives on the specific role of specialist palliative care services, and when they should be introduced for patients with NMRD.

### The presence of a model for palliative care delivery in NMRD

A model of palliative care for patients with NMRD can help to ensure they receive appropriate palliative care when it is needed. This was perceived by participants in NI:

*“Well there is the ELCOS (End of Life Care Operational System) Model for palliative care used for respiratory palliative care and that is regional and it moves from early to late phase and tells you what the patient should be getting at each stage. And I suppose with the model everything is there and you know what’s available and what you should be doing and us a team can look and see what we need to be doing.”* (RN2, Respiratory Nurse Specialist, p14).

Within the ROI however participants highlighted that there was not a regional model for palliative care that could help to guide the delivery of palliative care to patients with NMRD:

*“We don’t have a model for either delivering palliative care or respiratory care and I suppose we should.”* (RN5, Respiratory Nurse Specialist, p8).

## Discussion

The present study highlighted carers’ perceptions that patients with NMRD wanted access to specialist palliative care services however service availability was often uncoordinated and varied. Research has highlighted this previously amongst patients with ILD [35–37] and COPD [38]. The present study however additionally discovered that patients with bronchiectasis also experienced a lack of involvement from specialist palliative care providers. The integration of early specialist palliative care can help alleviate the symptoms experienced by patients with COPD and ILD [39].

Prognostic uncertainty experienced by HCPs impacted on the referral of patients with NMRD to specialist palliative care services. Crawford et al. [40] discovered that often referrals to specialist palliative care services were linked to how long a patient with COPD had left to live, rather than their holistic symptom needs. This implies that HCPs may be reluctant to refer patients for specialist palliative care input if they are unsure how their disease is progressing. Healthcare professionals must make referrals to specialist palliative care based on a needs assessment of the patient’s bio-psychosocial symptoms. This should not just be based on when the patient will die, to ensure optimal management of their holistic needs. By predicting needs instead of prognosis, patients with NMRD could be delivered more proactive palliative care [14, 41].

Research comparing access to specialist palliative care services between patients with NMRD and lung cancer, internationally and within the UK, indicated that patients with COPD and ILD have less access to specialist palliative care services than patients with lung cancer [7, 8, 42]. It has been previously suggested that this may be a result of HCPs associating palliative care with malignant disease [7, 8]. Present findings added a novel perspective by further highlighting that this is also a misconception associated with bronchiectasis, not just COPD and ILD. If HCPs are unaware of the role palliative care has in the care of patients with NMRD then this will impact on the services this client group receives.

Healthcare professionals additionally indicated that patients with COPD may more commonly receive generalist and specialist palliative care than those with a diagnosis of ILD or bronchiectasis. Previous literature acknowledged the importance of palliative care for patients with bronchiectasis [43, 44] and ILD [35, 36]. Bajwah et al. [35] also highlighted that the important role of palliative care in ILD was not always recognised in the UK as it was not a malignant disease. Within the present study however, it was additionally evidenced that inequalities related to the recognition of the role of palliative care may also be seen amongst different types of non-malignant disease, and not just between cancer and non-cancer diagnoses. Patients with COPD are less likely to get referred for specialist palliative care than patients with other non-malignant disease such as heart failure and severe dementia [38]. It is important that HCPs recognise that the provision of palliative care to patients with NMRD must be based on need [14], to ensure the effective provision of palliative care that is responsive to the patient’s symptom complexities.

Focus group findings presented various views on how future palliative care should be delivered to patients with NMRD. Some HCPs highlighted that specialist palliative care should be provided to all patients with NMRD. International guidance has recognised that palliative care should be delivered by generalist HCPs, and specialist palliative care providers should be involved in a patients care when their symptoms become increasingly complex [21]. However research conducted in the UK advocated the integration of early specialist palliative care for patients with ILD and COPD, and provided evidence that it helped improve the mastery of breathlessness experienced by these patients [39]. Guidelines within the UK have also recommended the introduction of specialist palliative care services for patients with ILD from the point of diagnosis [18]. Policies specific to bronchiectasis however do not provide guidance on the introduction of these services [18, 22].

In line with international views [45], and previous research [46], many HCPs expressed that specialist palliative care should only be introduced when generalists find it difficult to manage the bio-psychosocial symptoms of patients with NMRD. Policy [19], research [47] and international guidelines [21, 48] have advocated the efficacy of specialist palliative care services being introduced when patients with advanced COPD and ILD develop complex symptoms. However there is a lack of consistent guidance for HCPs involved in the care of patients with NMRD, regarding the palliative service provision required by this client group.

For some HCPs, within the present study, the reasoning behind championing the role of specialist palliative care for patients with NMRD was due to its perceived impact on reducing unplanned hospital admissions. Previous research highlighted that significant decline in admissions for COPD exacerbations may have been aligned with specialist palliative care services providing palliative care to this client group [49]. The present study concurs with these findings as some HCPs felt that patients with NMRD who received specialist palliative care were less likely to attend accident and emergency departments, or have unplanned admissions to hospital. This may be due to perceptions that patients with NMRD who have specialist palliative care teams involved in their care will have more effective symptom management [39], and can therefore be effectively managed within their preferred place of care.

**Fig. 3:**
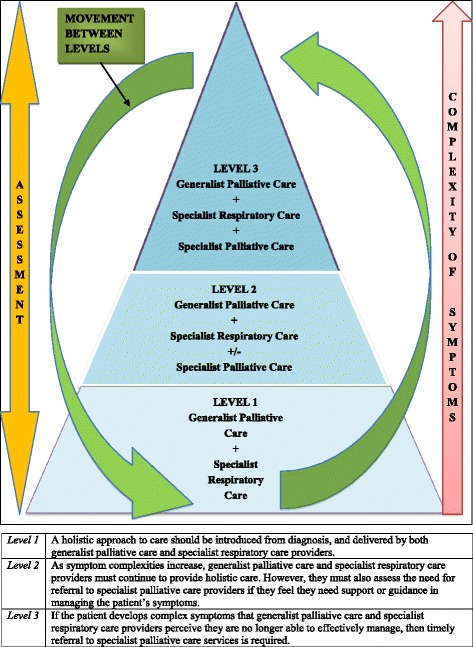
Model of palliative care for patients with non-malignant respiratory disease

Previous research has identified varying potential models of palliative care for patients with ILD and COPD [19, 20, 39, 46]. However, previous policy documents and research have been void of guidance regarding a model of palliative care for patients with bronchiectasis. Additionally, if patients’ holistic symptom needs were treated rather than their prognosis or diagnoses anyone with a life limiting illness would get help when needed e.g. patients with bronchiectasis or other rare diseases that will rarely be thought of as needing palliative care. Findings from the present study highlighted that HCPs might have benefited from a model of care to guide the provision for palliative care for patients with NMRD that emphasised the importance of introducing holistic care early in the disease trajectory, and ensuring it is needs based. Figure 3 demonstrates a potential model of palliative care for patients with NMRD derived from the findings of the present study. This model proposes three levels of palliative care for patients with ILD, COPD and bronchiectasis whilst advocating the continued holistic assessment of patient need. Level one advocates a holistic approach to care which is introduced from diagnosis of NMRD. This care should be delivered by both generalist palliative care and specialist respiratory care providers. As symptom complexities increase, level two suggests that HCPs must additionally assess the need for the introduction of specialist palliative care services. The proposed model indicates that this decision should be based on HCPs feeling they need further support or guidance to manage the patient’s symptoms. If the patient develops complex symptoms that generalist palliative care and specialist respiratory care providers perceive they are no longer able to effectively manage, then level three of the model proposes that timely referral to specialist palliative care services is necessary. The patient may move between levels at different points within their trajectory and this should be based on the assessment of their needs and symptom complexities.

With regard to future research, an interesting extension of this study would be adopting the Medical Research Council (MRC) framework for complex interventions [50] in order to develop and ratify the potential model of palliative care derived from the findings of this study. This would initially involve conducting a feasibility study with respiratory teams to explore the practicalities of implementing the proposed model of palliative care for patients with ILD, COPD and bronchiectasis. The next stage would be interventional research to provide in depth information regarding the implications of introducing a model of care into the palliative care of patients with NMRD. This stage would focus on testing the intervention based on the data gained from the feasibility study. This intervention study could be modelled on previous research that has explored the implementation of models of palliative care for patients with breathlessness [51], ILD and COPD [39] and lung cancer [52].

### Limitations

Eighteen HCPs were recruited into the study however the majority who participated in the focus groups were nursing staff (*n* = 13). Increased numbers of allied HCPs and medical staff may have provided more diverse perspectives. Only a small number of participants had cared for someone with a diagnosis of ILD (*n* = 4) or bronchiectasis (*n* = 1). Recruiting greater numbers from these disease groups may have provided further perspectives. Findings also only represented the perspectives of carers and HCPs and not the patient’s own perspective. It is also recognised that bereaved carers were recruited through the RNS which makes the sample less representative of patients with NMRD who do not receive RNS care.

## Conclusions

The management of patients with NMRD is complex and challenging with a clear need for a stronger and more integrative model of practice, which incorporates palliative care in a responsive and dynamic way. This study has reinforced the importance of proactive palliative care that identifies the needs of the individual patient and is not influenced by their prognosis. Future care to patients with NMRD must also acknowledge the important role of palliative care and generalist palliative care providers must have access to specialist input and advice when needed. This study also adds a novel perspective in identifying a potential model of palliative care for NMRD that may enhance future holistic care for this client group. The feasibility of integrating this model into clinical practice requires further exploration.

## Additional files

Additional file 1: Box 1. Interview Guide. (DOCX 12 kb)

Additional file 2: Box 2. Focus Group Guide. (DOCX 12 kb)

## Abbreviations

COPD: Chronic obstructive pulmonary disease
HCP: Healthcare professional
ILD: Interstitial lung disease
MDT: Multidisciplinary team
MRC: Medical Research Council
NI: Northern Ireland
NMRD: Non-malignant respiratory disease
RNS: Respiratory nurse specialist
ROI: Republic of Ireland
UK: United Kingdom
WHO: World Health Organisation
