# Supplementary material for: The provision of generalist and specialist palliative care for patients with non-malignant respiratory disease in the North and Republic of Ireland: a qualitative study
Source: BMC Palliat Care. 2017 Jul 11;17:6. doi: 10.1186/s12904-017-0220-1 (PMC5504568; doi:10.1186/s12904-017-0220-1)
Supplement: Supplementary file 1 — Box 1. Interview Guide. (DOCX 12 kb) [file 12904_2017_220_MOESM1_ESM.docx]

**Box 1.** Interview Guide

| **Opening Statement**  Can you tell me about the care your relative received towards their end of life?  **Areas to Be Covered (Prompts in brackets)**   1. One area I would like to talk about is what services and support did your relative experience from the health care profession in their last year of life? (What, who, efficiency, sufficiency, any further needed) 2. Another area I wish to discuss is what services and support did you experience from the healthcare profession while caring for your relative in their last year of life? (What, who, efficiency, sufficiency, any further needed) 3. I would like to discuss the symptoms your relative experienced in their last year of life and how these symptoms were managed. (Sufficiency of information, staff knowledge and expertise, quality of symptom management, emergency contacts for symptom management) 4. Can you tell me about when and where your relative died and if you felt this was their preferred place to die. (why they died here, anything further could have been done to facilitate preferred place to die) 5. Can you tell me about what support you received after the death of your relative, if any, and how did you feel about this support? 6. Thinking about your experience caring for your relative, do you think there was any service or support that could have been provided that would have been beneficial for you or your relative? |
| --- |
