# Supplementary material for: The provision of generalist and specialist palliative care for patients with non-malignant respiratory disease in the North and Republic of Ireland: a qualitative study
Source: BMC Palliat Care. 2017 Jul 11;17:6. doi: 10.1186/s12904-017-0220-1 (PMC5504568; doi:10.1186/s12904-017-0220-1)
Supplement: Supplementary file 2 — Box 2. Focus Group Guide. (DOCX 12 kb) [file 12904_2017_220_MOESM2_ESM.docx]

**Box 2.** Focus Group Guide

| 1. To start, could anyone tell me about the palliative health care provision that is currently available for people with non- malignant respiratory disease and their carers, both specialist and generalist? 2. What do you feel would make for a high standard of pall care post discharge for these patients and carers? 3. Can you tell me how well generalist palliative care and specialist palliative care services are organized and resourced both in the hospital and community setting? 4. Can you tell me how effective are the lines of communication between the HCPs involved? (Especially between generalist palliative care and specialist palliative care ) 5. Can you tell me how well generalist palliative care and specialist palliative care services are accessed by this client group both in the hospital and community setting? 6. How do you perceive patients and their carers understand of the meaning of palliative care? 7. What do you perceive as good palliative care? 8. What are the barriers and facilitators of good palliative care? 9. Any suggestions for improvement? 10. How do you feel good generalist palliative care compares with specialist palliative care? 11. How do you perceive the generalist palliative care and specialist palliative care services available to patients with a diagnosis of either bronchiectasis or interstitial lung disease (ILD) and their carers, compares to the services available to those with a diagnosis of COPD? 12. How do you perceive the generalist palliative care and specialist palliative care services available to NMRD patients and their carers in rural areas compares to the services available to those in urban areas? 13. Can you tell me about any Models of Palliative Care that are used at present to guide the provision of palliative care? 14. How do you perceive the role of the Healthcare Support Worker in delivering palliative care to patients at home? 15. Can you tell me how you perceive how generalist palliative care and specialist palliative care providers communicate with NMRD patients and their carers? 16. Can you tell me your perceptions about how the information needs of carers and patients are met? 17. Can you tell me about the barriers and facilitators in relation to communicating with patients and carers? 18. Can you tell me how you feel about how effective discussions around end-of-life and prognosis with patients and carers are? 19. What are the barriers involved in having these conversations? 20. Any suggestions for improvement? 21. How effective do you feel HCPs are at taking into consideration the wishes of the patient and their family? |
| --- |
